# Choice of Nanoparticles for Theranostics Engineering: Surface Coating to Nanovalves Approach

**DOI:** 10.7150/ntno.89768

**Published:** 2024-01-01

**Authors:** Rajendra Prasad, Kaliaperumal Selvaraj

**Affiliations:** 1 Interventional Theranostics & Multimode Imaging Lab, School of Biochemical Engineering, Indian Institute of Technology (BHU), Varanasi 221005, Uttar Pradesh, India.; 2 Nano and Computational Materials Lab, Catalysis and Inorganic Chemistry Division, CSIR National Chemical Laboratory, Pune 411008, India.; 3 Academy of Scientific and Innovative Research (AcSIR), Ghaziabad, 201002 India.

## Abstract

Surface engineered nanoparticles (metallic and nonmetallic) have gained tremendous attention for precise imaging and therapeutics of cell/tumors at molecular and anatomic levels. These tiny agents have shown their specific physicochemical properties for early-stage disease diagnosis and cancer theranostics applications (imaging and therapeutics by a single system). For example, gold nanorods (AuNRs) demonstrate better photothermal response and radiodensity for theranostics applications. However, upon near infrared light exposure these AuNRs lose their optical property which is characteristic of phototherapy of cancer. To overcome this issue, silica coating is a safe choice for nanorods which not only stabilizes them but also provides extra space for cargo loading and makes them multifunctional in cancer theranostics applications. On the other hand, various small molecules have been coated on the surface of nanoparticles (organic, inorganic, and biological) which improve their biocompatibility, blood circulation time, specific biodistribution and tumor binding ability. A few of them have been reached in clinical trials, but, struggling with FDA approval due to engineering and biological barriers. Moreover, nanoparticles also face various challenges of reliability, reproducibility, degradation, tumor entry and exit in translational research. On the other hand, cargo carrier nanoparticles have been facing critical issues of premature leakage of loaded cargo either anticancer drug or imaging probes. Hence, various gate keepers (quantum dots to supramolecules) known nanovalves have been engineered on the pore opening of the cargo systems. Here, a review on the evolution of nanoparticles and their choice for diagnostics and therapeutics applications has been discussed. In this context, basic requirements of multifunctional theranostics design for targeted imaging and therapy have been highlighted and with several challenges. Major hurdles experienced in the surface engineering routes (coating to nanovalves approach) and limitations of the designed theranostics such as poor biocompatibility, low photostability, non-specific targeting, low cargo capacity, poor biodegradation and lower theranostics efficiency are discussed in-depth. The current scenario of theranostics systems and their multifunctional applications have been presented in this article.

## Introduction

Nano(bio)technology has remarkable impact on nanomedicine especially cancer theranostics. Nanoparticles of metals (gold, silver, iron, copper, bismuth), self-assemblies of biomolecules (viz., liposomes, micelles, dendrimers, etc.), various carbon-based nanomaterials (carbon/graphene quantum dots, nanoflakes, nanolayers, nanosheets, etc.), recently replacing several fluorescent organic dyes and their composites are some of the recent best examples for various biomedical systems including drug delivery systems, image contrasts and combination therapeutics. Complicated synthesis routes, poor biocompatibility, low photo-stability, non-specific targeting, low cargo capacity, poor biodegradation and lower theranostics efficiency are the general major hurdles with these materials. Rapidly emerging advanced systems for biomedical applications, namely multifunctional theranostics agents are designed to support multiple tasks in diagnosis and advanced therapy. Developing a successful one for the combined therapeutic approaches of cancer viz., chemo, photothermal and ablation etc. along with diagnosis is a crucial necessity at this juncture. Among the above liposomes, PEGylated silica-gold nanoshell (AuroShell) and silica-quantum dots (C dots-silica composite) are few that have been approved for clinical trials. The recent recognition of liposomes, gold and mesoporous silica (MS) by US FDA as safe material for human trials is another milestone for in this area. The present article deals closely with this area and involves several of the mentioned materials.

Few positive attempts in designing such theranostics agents during the course of degree have been presented here. This includes multifunctional nanohybrid systems that can support (a) diagnosis (X-ray CT and fluorescence), (b) chemotherapy (using doxorubicin a popular anti-cancer drug) and (c) photothermal therapy/ablation (using NIR laser) along with various features such as targeted, triggered and bio-responsive drug release etc. Designing such systems through simple, rapid, and ambient recipes is an apparent demand. Very recently attempts have been made in this area and with reports displaying major challenges involved in this. Main task is to ensure structurally embedding several individual components and yet to assure a synergistic and better controlled performance. Reproducibility associated with such systems while scaling up production is another important challenge. In this context, the main objective of the research problem addressing through this article is (i) to design multifunctional hybrid nanomaterials (ii) to successfully embed various functional components (for applications viz., targeting, contrast and therapeutics) in single system and (iii) to ensure their performance for cancer theranostics. This includes the use of various components such as mesoporous silica, gold nanorods, carbon quantum dots, NIR responsive graphene oxide flakes, liposomes etc. Several positive attempts are briefly described in different sections along with complete scientific details.

An overview of nanoparticle research for cancer theranostics (diagnosis and therapeutics) along with the statement of concerns and research developments have been discussed here. A section highlights the importance of plasmonic theranostics viz., gold nanorods (GNRs) for cancer diagnosis and photothermal therapy. A surfactant named cetyltrimethylammonium bromide (CTAB) is known as a structure directing agent for stabilizing GNRs, however, is a limitation in its biomedical application due to its toxicity. To overcome the biocompatibility and stability concerns, silica layers coating on GNR's surface has been utilized and even tested in *in vivo* models. This core-shell nanotheranostics exhibit improved contrast ability, good circulation time, targeted combination therapeutics and other multifunctionality. To the best of our knowledge and expertise, bare porous silica, especially mesoporous silica (MS) has been reported for drug delivery applications with high cargo capacity (more than 50 %) and surface area (900-1000 m^2^/g). However, premature leakage of loaded cargo is the major issue of such hybrid delivery system. Our group has resolved this problem by using bio-responsive fluorescent carbon dots as nano-gates on the exterior surface of mesoporous silica nanoparticles. This nano-gated platform performs theranostics response with intracellular tracking of cellular components through fluorescence emission and its bio-responsive drug release in cancer mimicked environment. However, in nanotheranostics, low biocompatibility and slow biodegradation of inorganic systems are major concerns. Recently, a few groups including us have designed biodegradable systems based on soft assemblies known as liposomal nanoparticles and their surface engineering. Overall accomplishments and potential future scopes of the research area have been summarized in this review.

## Engineering and choice of nanoparticles in theranostics

Manipulation of matter at atomic, molecular and supramolecular scale to create materials with remarkably new properties is a rapidly expanding area of materials engineering 1-4. Surface engineering science involves an understanding of interconnect between synthesis routes, structural design and properties of the nanoparticles of nanohybrid materials 3. Depending upon the property, a well-designed multifunctional material could have promising applications in the areas spanning chemistry, physics, biology and engineering 3,5,6. To date, functional hybrid nanomaterials have been applied in various fields especially for imaging and therapeutics of cancer shown in **Table 1** 7-13. One of the most significant and impactful applications of nanomaterials is in the area of nanomedicine. So far, several nano sized hybrid/composite materials (nanohybrids/nanocomposites) are being applied as diagnostic or therapeutic probes with their tremendous impact on human health 14,15. Further, a single hybrid nanostructure capable of multi-model diagnostics and combined therapies (chemo-photothermal therapy, chemo-photodynamic, photothermal-photodynamic therapy etc.) is an area of recent development with possible significant outcome on health care 15-24. Integration of multiple components with multiple functions at nano level produces hybrid nanostructures that allows the above interest of application 18,22,24.

In the context of human health care, imbalances in genetic and recently evolving environmental factors increase the possibilities of multifactorial complex diseases such as cancer, a leading cause of mortality in the modern world 2. Cancer is understood in a better way from the genetic, molecular, and cellular biology viewpoints requiring new strategies of diagnosis and treatment. In cancer, nanotechnology-based medicine has great potential in the area of new drug delivery systems, multifunctional diagnostics and as therapeutic agents 7,16.

Comprehensively used various nanoparticles (NPs) for this have overall dimensions less than several hundred nanometers and are comparable to large biomolecules, such as enzymes, receptors and antibodies 16-20. On account of this specific physical property, these nanoparticles (NPs) exhibit excellent interactions with biomolecules present on the exterior and interior environment of cancer cells. However, generally, nonspecific targeting nature, multiple heavy dose requirements of contrast agents and drugs, poor water solubility, rapid clearance and time-consuming planning are limiting their applications in today's diagnosis and therapies 25. In addition, traditionally used chemotherapy and radiation therapy treatments are less effective for cancer patients due to side effects from heavily used radiation and drug molecules 26-29. As solution to above, nanoparticles designed with combined diagnostic and therapeutic abilities is a recently developing concept for cancer detection and targeted therapies 26,28. Performing high efficiency of both imaging and therapeutic functions by a single hybrid material is the major advantage of nanosized multifunctional systems and consequently various research efforts have been devoted towards this.

## Nanotheranostics and advantages

**Figure 1:**
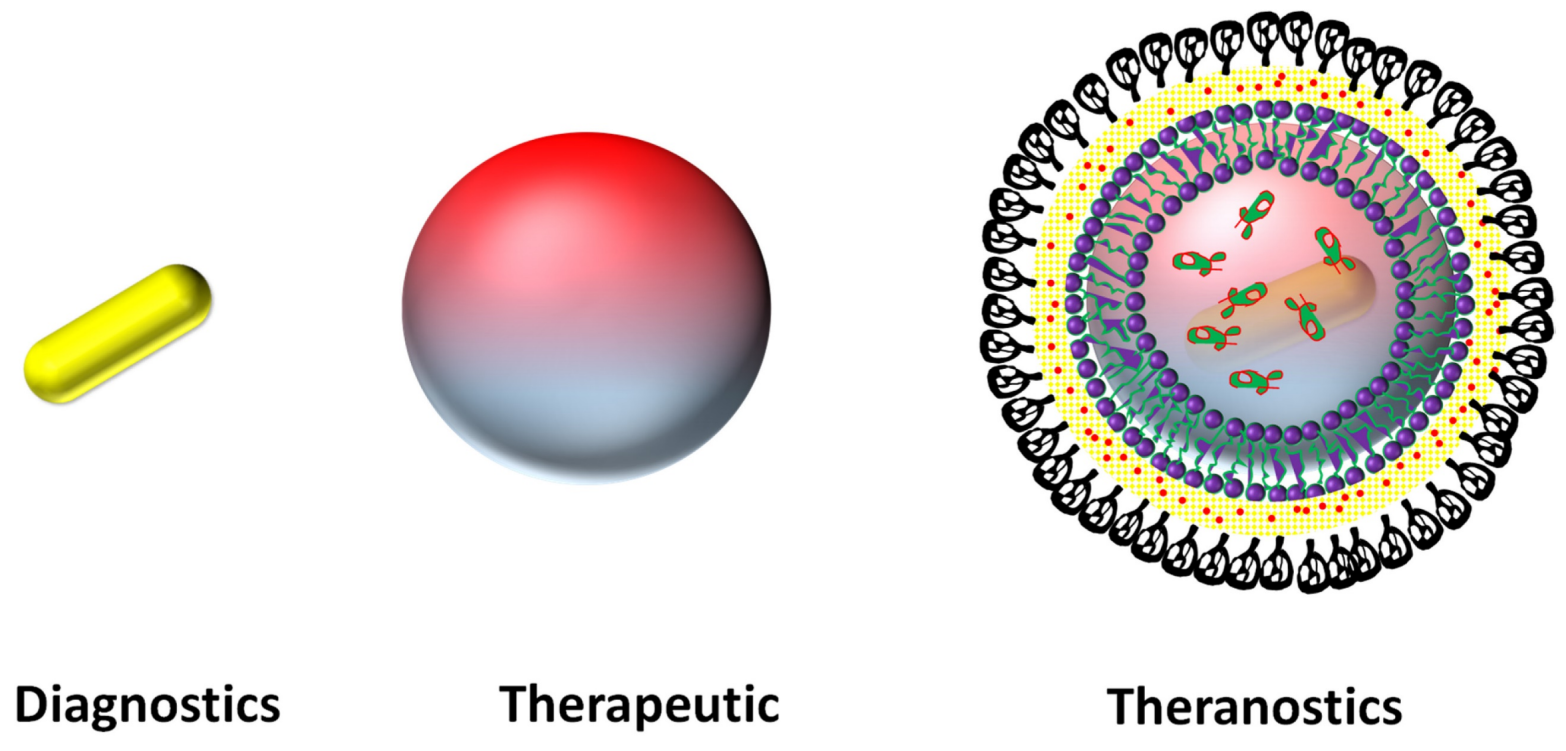
Representative nanoparticles based targeted theranostics design.

In 2008, the major challenges and opportunities within newly designed medical products are reported by U.S. Food and Drug Administration (FDA) 30. On the other hand, John Funkhouser, the Chief Executive Officer of PharmaNetics, introduced the term “Theranostics” (meaning diagnostics and therapeutics by a single system) for the first time in 1998 as a concept. Furthermore, the primary objective of theranostics is to be able to monitor the response of treatment, effect of drug efficacy and safety for healthcare systems. Recent advances in nano biomedicine have expanded the ability to design and fabricate multifunctional nanoparticles that combine targeting, therapeutic and diagnostic functions in a single nanoplatform 22-24,31,32 in the specific that integrates, the combination of contrast/diagnostics (molecular imaging) and therapeutics agent (molecular targeted treatment “all-in one” **Figure 1**) 32. Imaging and therapeutic modality have their unique strengths for medical diagnosis and treatment. Imaging has advantages for diagnosis from two or more imaging modalities in a single platform that provides overall structural and molecular information 33,34. Similarly, the multimodality in therapy also shows their significant advantage on cancer cell/tumor elimination 23. Combined modalities of diagnostic and therapeutic agent in a single system at nanoscopic level is newly emerging as “nano-theranostics” an urgent need for nanomedicine.

Their major and visible advantages are high water solubility, specific optical properties, good biocompatibility, high theranostics efficiency, easy to functionalize with biomolecules (capping agent/targeting ligands), high cargo capacity, smooth circulation, localized binding ability and high tumor accumulation 29,31-34. Image guided stimuli triggered targeted cancer therapy could be a new rapid advance for theranostics. The distinct advantage of these theranostics is to provide localized and detailed information and therapy in specific sites of diseases 35-39. However, challenges such as optimal circulation, biocompatibility, biodegradation and excretion remain as major concern and are currently being addressed 40-50.

## Components of theranostics system

A successful design of a theranostics system essentially needs understanding of various functions and the components that perform them. Nanosized imaging (contrast/emissive media) and therapeutic agent in a single system is a recent development in nanomedicine 20-24,32,35,39. Today, several diagnostic techniques such as positron-emission tomography (PET), X-ray computed tomography (X-ray CT), optical coherence tomography (OCT), magnetic resonance imaging (MRI) etc. are routine procedures in hospitals due to the unparalleled and complementary details that they can provide about the interior body 35,36. These techniques are becoming progressively a significant piece of the diagnostic portfolio available to physicians that helps in the understanding the specific location of tumor in animal or human body. In order to facilitate the understanding of healthcare practitioners on the abnormalities of a diagnostic image, contrast agents are often employed to create visible changes in the resulting image after interacting with the incident radiation 35. The above imaging modalities are heavily dependent on the choice of contrast agents and thus this makes one important function/component of theranostics system. The development of nanosized novel and improved contrast agents has significant impact to this field further improvement on their contrast ability, biocompatibility, targeting ability and specific bio-distribution etc are intensively being investigated 35.

Among all, the X-ray computed tomography (X-ray CT) technique provides detailed three-dimensional (3D) anatomic information with high resolution and good brightness 51. X-ray CT is one of the commenly used imaging techniques due to its wide availability, high efficiency and quick response. This technique is characteristically accomplished by stimulating multiple X-rays from different angles to form slices which can further be composited collectively to form a detailed set of cross-sectional image 52. In this context, iodine and gadolinium based contrast agents are commonly used due to atomic number and X-ray attenuation 35. However, these contrast agents suffer from many problems including short circulation half time (<10 min. for conventional iodinated contrast), nonspecific bio-distribution, rapid excretion and renal toxicity, poor contrast ability, high dose requirement (~ 100-400 mg/mL concentration) 35,51,52. Hence, in recent years, various nanosized contrast agents have been developed which are able to overcome many of these shortcomings. Nano-formulations of bismuth, barium, gadolinium, dysprosium, ytterbium, tantalum, gold nanoparticles, etc. with high atomic number and X-ray absorption coefficient have been investigated for multimodal diagnosis 35. Various fluorescent contrast agents such as organic dyes, NIR responsive quantum dots (metal and graphene/carbon dots) for emissive diagnosis are also designed in recent years 53,54.

**Figure 2:**
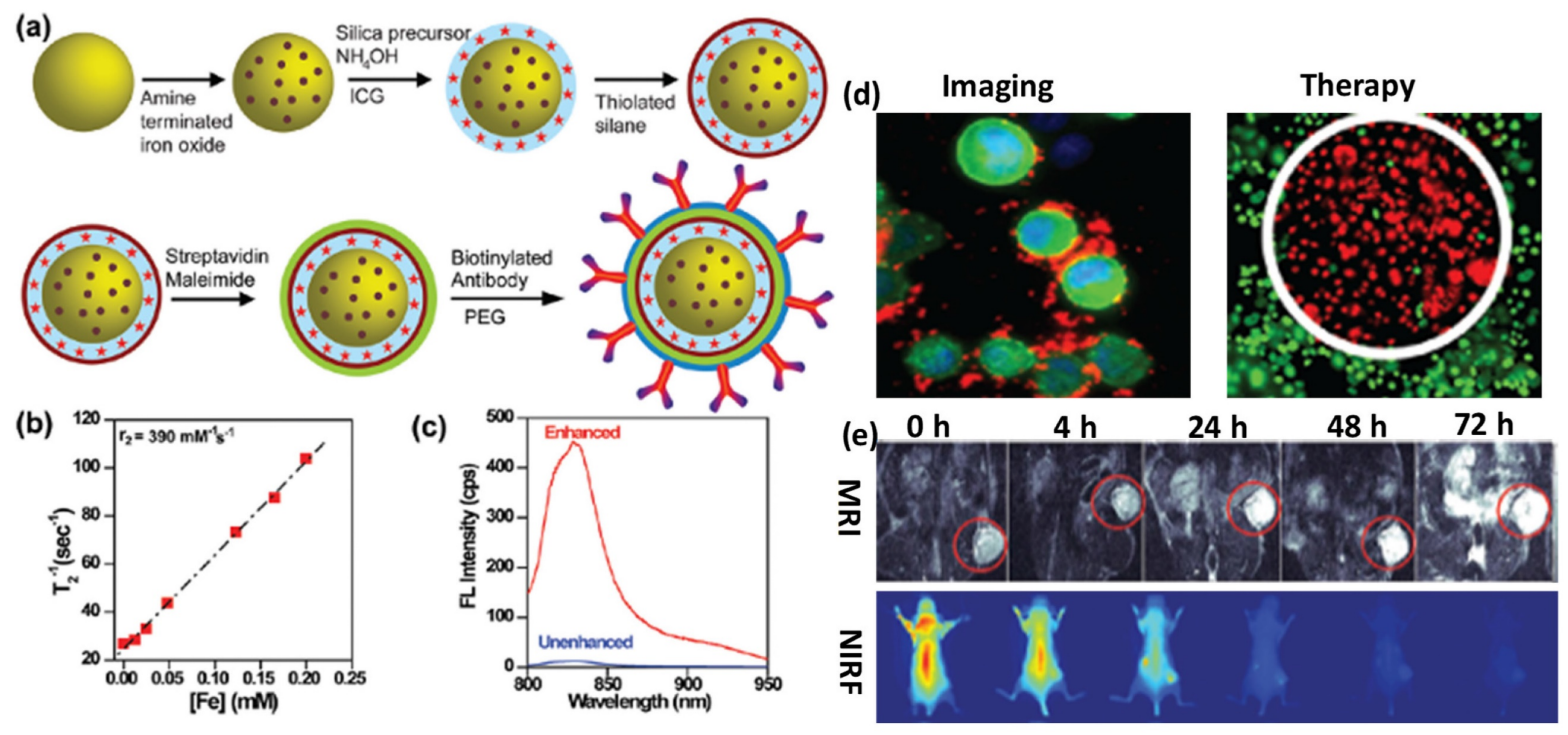
(a) Schematic of surface engineered nanotheranostics, (b) Spin-spin relaxation rate, (c) fluorescence (FL) spectra of ICG dye tagged engineered nanotheranostics, (d) in vitro cancer cell imaging and therapeutics and (e) in vivo tumor imaging followed by multimode imaging modalities. Reproduced with permission from Acc. Chem. Res., 2011, 44, 936 published by American Chemical Society.

Another major component of theranostics system is a carrier system that transports the contrast agent and/or therapeutic agent to the interested specific site with the help of a targeting ligand (**Figure 1 and Figure 2**). A number of carrier systems are designed with their high cargo loading capacity such as inorganic porous hollow spheres, mesoporous silica nanoparticles, magneto-plasmonic nanocapsules, gold nano shells, liposomal, polymeric and graphene oxide nanoparticles, etc 35,55-61. The combination of both imaging agent and carrier in one system fulfills the fundamental criteria of nanotheranostics. These hybrids facilitate easy functionalization of targeting ligands on its surface to have longer circulation (at least 24 h), specific targeting and bio-distribution 23,40.

**Figure 3:**
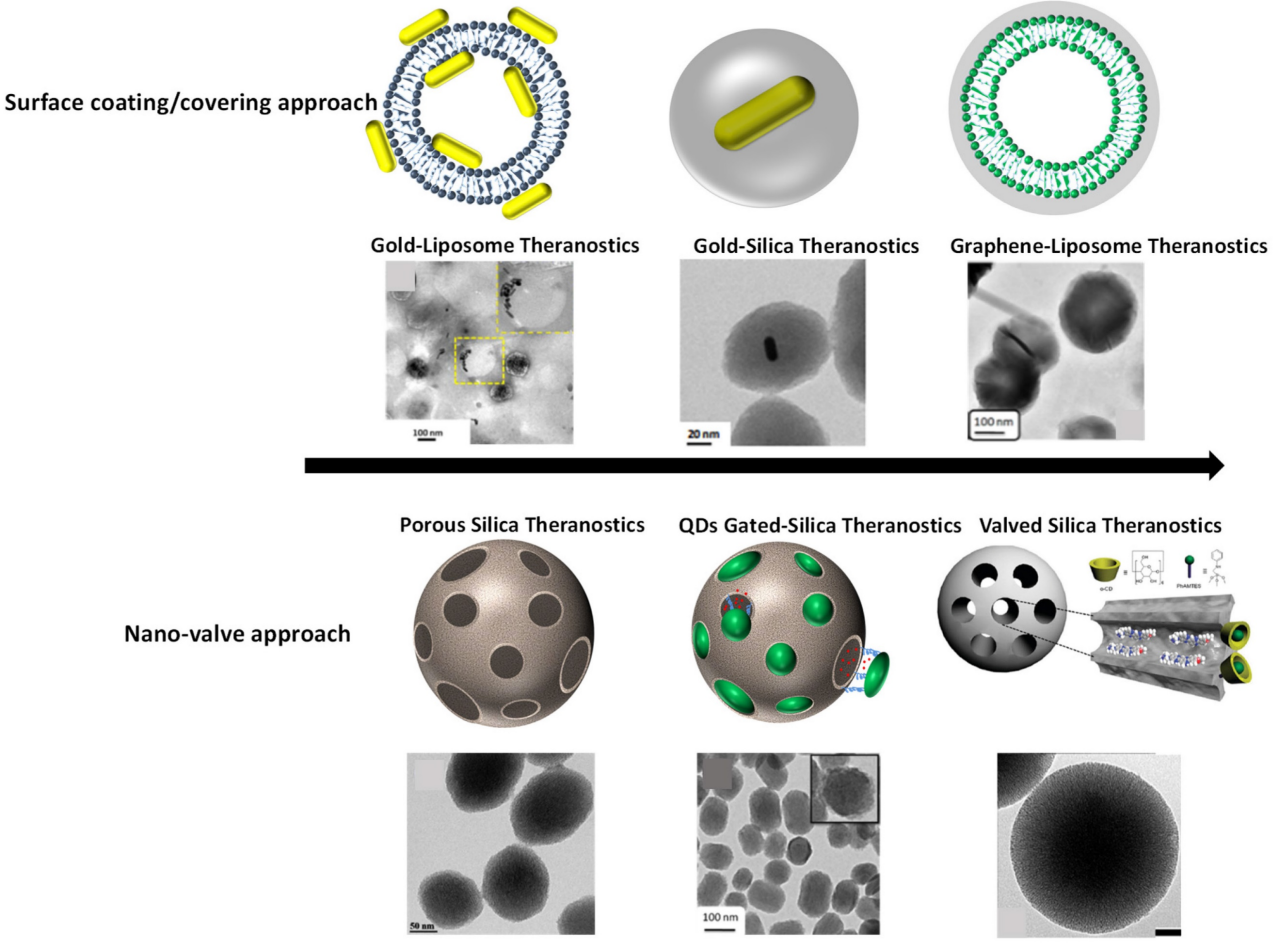
Schematics of surface engineered nanotheranostics. (Top) surface covering/coating of nanoparticles viz., liposomal surface covering with gold nanorods and graphene oxide, gold nanorods surface coating with silica shell, reproduced with permission from Bioconjugate Chem. 2018, 29, 5, 1510, Bioconjugate Chem. 2018, 29, 12, 4012, ACS Appl. Bio Mater. 2019, 2, 8, 3312 published by American Chemical Society. (Bottom) mesoporous silica nanoparticles and their pores are valved/or gated with nanodots of carbon reproduced with permission from ACS Appl. Bio Mater. 2021, 4, 2, 1693 published by American Chemical Society, Nanoscale, 2016, 8, 4537 published by Royal Society of Chemistry and J. Phys. Chem. C 2011, 115, 40, 19496 published by American Chemical Society.

External stimuli responsive combined therapeutics viz., chemo-photothermal therapy, chemo-photodynamic therapy, photothermal-photodynamic therapy are other important capabilities of theranostics systems. Combining above mentioned multiple therapies into a single system are more effective and significant for cancer treatment. Various nanohybrids/nano-composites such as gold nanorods embedded porous silica, dye loaded mesoporous silica, MoS_2_ nanohybrids, NIR light responsive and thermal responsive graphene-gold nanohybrids/ nano-composites etc. have been explored for combined therapeutic applications due to their high rate of light-to-heat conversion, drug loading capacity, a large amount of reactive oxygen species generation, etc 8,12,17,18,22. Imaging agent integrated therapeutics platforms have been engineered by surface coating/core-shell and surface conjugation. In the case of porous hybrid theranostics systems, premature leakage of loaded cargo is always a critical concern. To prevent the premature release of therapeutics molecules before reaching them to the target site, nano-valved approach has been proposed recently. This surface coated and nano-gated engineered theranostics systems satisfy the theranostics requirements with several advantages as shown in **Figure 3**. Next generation of combined therapy will help in following: (i) reduced side effects, (ii) enhanced efficacy of therapeutic outcomes, (iii) improved survival and quality of life of cancer patients.

## Current scenario of integrated nano-imaging and therapeutic agents

Theranostics with additional functions are progressively getting popular especially with combined therapeutic abilities 18,22. From molecular understanding to clinical trials, few of them have been translated. Some are on in vivo studies 22-25. Fabrication of nano sized theranostics systems is highly interesting for medical scientists. However, large scale production through rapid process, aqueous solubility, high cargo capacity, localized controlled release, biocompatibility, circulation, and specific targeting ability are the major concerns and also current demands for any types of theranostics 61-63. Designing bio-responsive, degradable and targeted nanotheranostics systems are though of current demand for nanomedicine yet remain as major challenges. The designed theranostics systems should be able to show their best performance at specific sites in cancer cells or at tumor environment. Thus, real time monitoring of delivered nanotheranostics in cancer mimicked environment can be made possible to provide critical information about specific diagnostics and therapeutic efficiency. So far, various nanoparticles such as metal (Au, Ag, Fe, WS_2_), liposome, polymers, mesoporous silica, silica coated gold nanorods, graphene quantum dots, nano-gated porous silica, up-conversion luminescent materials and many more have been used for multifunctional theranostics applications 34,64. A number of studies have confirmed their potential usage for theranostics, and various formulations of these nanoparticles are already in the preclinical and clinical stages of development. For example, PEGlated silica-gold nanoshell (AuroShell) as a photothermal agent, silica-quantum dots (C dots-silica composite) for molecular imaging, PEGlated liposomal doxorubicin for ovarian cancer treatment, etc., are used for human trials for the first time 65-69. Several other nano sized formulations are under clinical trials and many more are currently being evaluated at the preclinical level for cancer diagnosis and treatment 69. However, several properties of these theranostics systems need to be understood such as surface chemistry, dispersibility/solubility, stability, doses/concentration, biocompatibility, circulation time, pharmacokinetics, specific bio-distribution, and specific site binding ability prior to clinical applications. This enables them to become an important clinical weapon for cancer cure in the near future.

On other hand, traditionally used chemotherapeutic drugs and radiation therapy show heavy side effects on health 70. These conventional therapeutics require further improvements for better and fast treatment without any side effects. Photothermal therapy (PTT, photothermal agents which exhibit strong absorption in the near-infrared region (600-1100 nm), photodynamic therapy (PDT, oxidize targeted site via singlet oxygen which causes cell damage and death), thermal and magnetic hyperthermia have recently gained popularity as compared to the conventional radiotherapy and chemotherapy 22-24,34.

## Nanoparticles for theranostics engineering

As discussed above, various metallic and nonmetallic nanoparticles have been proposed and tested for theranostics applications. However, overall theranostics performance may be compromised while integrating imaging and therapeutics probes within a single system. It should be noted that sufficient chemical and physical protection of biological cargo molecules or payloads is possible by engineering safe carrier systems to deliver the loaded cargos to the desired target site. So far, several organic and inorganic carrier theranostics systems have been designed at nanoscopic level. Surface modifiers are attached to these systems and estimated to provide theranostics nanomaterials with additional properties such as preventing premature leakage of loaded cargos, long circulation time, barrier-penetrating ability and specific binding ability 28. Further, several organic-inorganic combinations are tried especially after 2005 and experienced a considered a rapid progress in nanomaterials design bringing about new opportunities for cancer diagnosis and treatment as shown in **Figure 3** 71-73.

Several cancer theranostics strategies are based on non-metallic, metallic, and composite nanohybrids 73. Fluorescent diagnosis and drug delivery are a major part of non-metallic systems. These systems are generally designed by carbon (top-down and bottom-up approaches) nanoparticles, organic dyes, soft materials (polymeric, micelles, surfactants etc.) and silica based (sol-gel methods) framework, etc 35. Due to aqueous solubility, good photoluminescence and stability, various formulations of carbon nanoparticles such as carbon quantum dots, graphene quantum dots, graphene oxide nano sheets, etc. are used as fluorescent diagnostics probe 74. In addition, various classes of mesoporous silica such as MCM-41, MCM-48, SBA-15, hollow silica spheres etc. highly explored for drug delivery applications 75. High surface area (700-1000 m^2^/g), highly ordered pores (2-50 nm in range), high cargo capacity (10-50 % of doxorubicin anticancer drug) and easy surface functionalization are the main features of these materials for biomedical applications 22-26,75.

On the other side, various metal nanoparticles like gold nanoparticles (many faces of gold, such as spheres, nanorods, nanocage, cubes, shell, nanoclusters, hollow), iron nanoparticles, silver nanoparticles are widely used for biomedical applications due to their unique characteristics such as highly tunable optical properties, contrast ability (high atomic number), specific surface plasmonic nature, good hyperthermia and photothermal efficiency 76,77. Moreover, these nanoparticles can efficiently convert incident light/radiofrequencies into heat 18,22. However, lack of chemotherapeutic efficiency of these nanoparticles limits their theranostics conditions 19. Further, the combination of non-metallic and metallic components in a single system is the better way to overcome the theranostics problems. The theranostics design depends on the interest of application. For example, gold nanorods-mesoporous silica nanohybrid 18 is suitable for CT based diagnosis and NIR responsive multifunctional cancer theranostics while nanovalved mesoporous silica nanohybrid 75 is fit for stimuli responsive triggered drug delivery with multimodal bio-imaging and iron oxide-silica composites are commonly used for MRI and magnetic hyperthermia for cancer theranostics etc.

**Figure 4:**
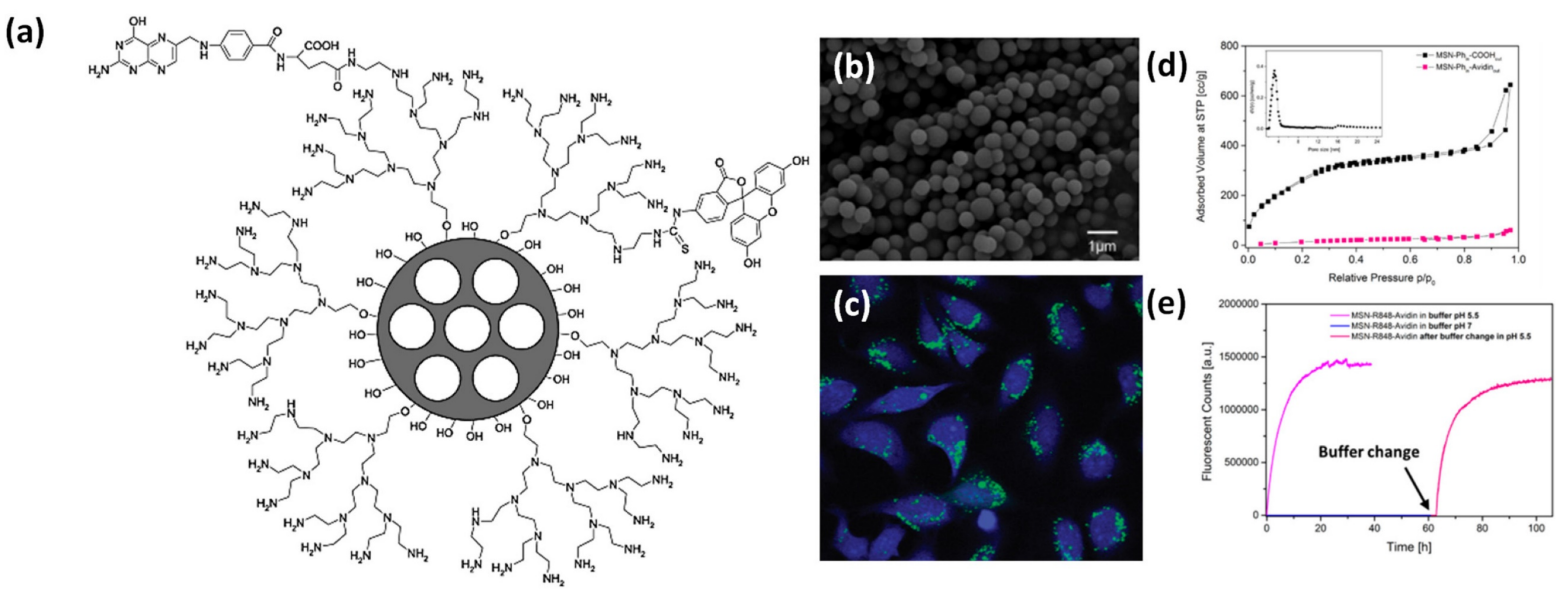
(a) Design of surface coated multifunctional mesoporous porous system and their (b) microscopic image and (c) HeLa cancer cell imaging, surface area isotherm and pore size distribution (inset) and time dependent fluorescent cargo release performance of conjugated silica-based nanoparticles. Reproduced with permission from ACS Nano 2009, 3, 1, 197 and ACS Nano 2021, 15, 4450 published by American Chemical Society.

To the best of our knowledge, nano sized metallic and non-metallic theranostics systems are limited due high cost of precursors, sophisticated and time-consuming synthesis process, poor control on premature cargo release, low product yield, poor stability, uncontrolled growth and particle size, low biocompatibility, nonspecific targeting ability and bio-distribution, poor image resolution, low cargo capacity, etc 34,35,38,47,63. In addition, several organic dyes and quantum dots have been studied for molecular imaging but due to poor photo blinking, photostability and biocompatibility, these are not recommended for *in vivo* or clinical applications but limited only for *in vitro* level 78. As a consequence, extensive efforts have been made to design new theranostics systems which can prevail over the above-mentioned limitations. Recent studies demonstrate that the mesoporous silica (MS, explored and declared as a safe material for human trials by US FDA) is recognized as a good cargo carrier due to its high surface area, pore size tunability, high cargo capacity, easy surfaces functionalization (both exterior and interior) with biomolecules and targeting ligands, chemical and physical inertness, good biocompatibility etc 79. Mesoporous silica based nanohybrids are highly recommended as a drug delivery system (DDS) due to high surface area, cargo capacity and easy surface modification shown in **Figure 4**. Clinical acceptance and trial (about 10) of silica-based hybrid nanoparticles ensure their safety profile in humans. It has been noticed that silica nanoparticles have shown good tolerability and better pharmacokinetic profile of hydrophobic drugs with no serious side effects. For example, surface modified silica hybrids viz., lipid coated silica particles have been used in 16 healthy adults for oral delivery of ibuprofen. These surface modified silica particles demonstrate 1.9 times higher bioavailability than bare silica nanoparticles. On the other hand, in another clinical trial (in 12 adults), lipoceramic-silica nanoparticles have demonstrated 3.5-fold higher bioavailability as compared to the commercially available simvastatin formulation Sandoz (ACTRN12618001929291). Overall, architectural control and surface decoration of these porous cargo carriers have brought new possibilities for controlled drug delivery in nanomedicine 75.

## Surface coated nanotheranostics systems

**Figure 5:**
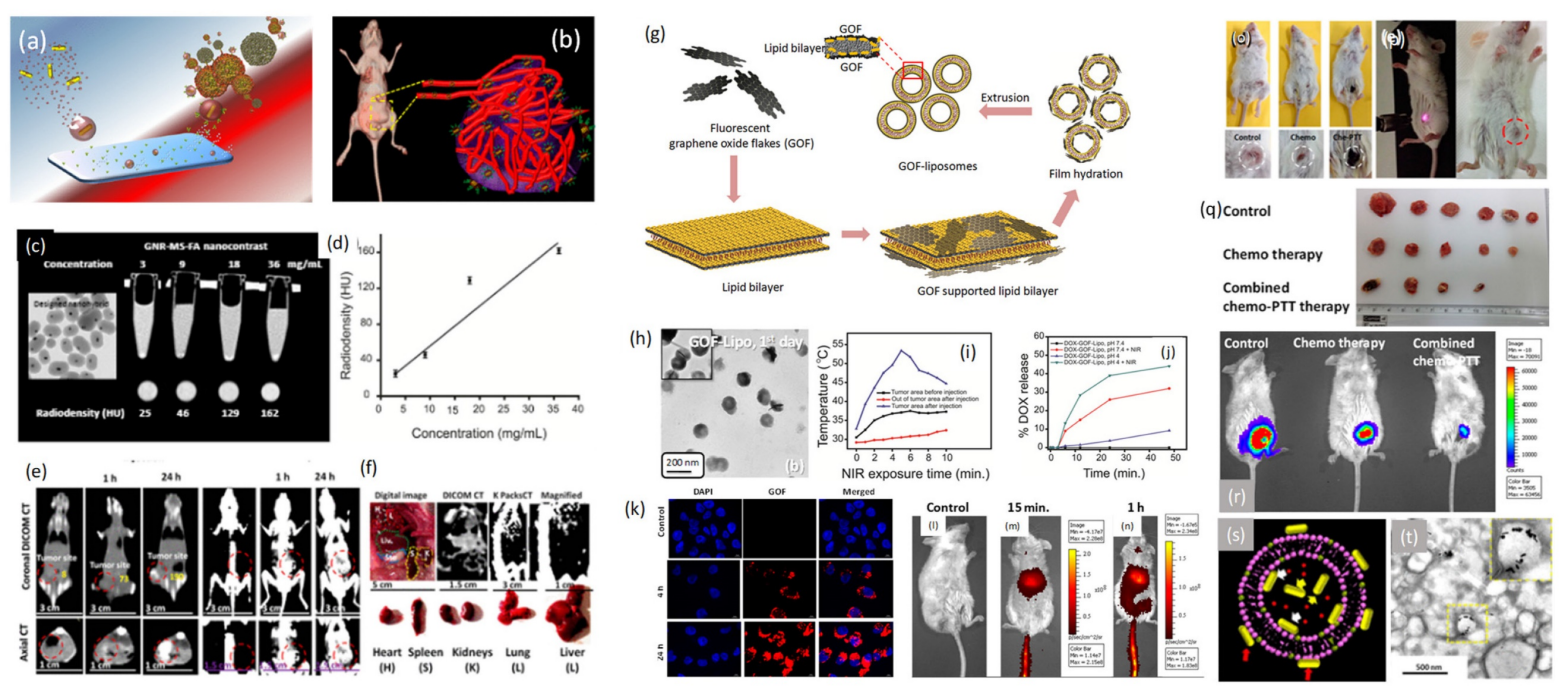
(a) Schematics of silica coated gold nanoparticles as surface engineered nanotheranostics, (b) uptake of nanotheranostics in solid tumor environment, (c, d) microscopic image of the designed nanotheranostics and their in vitro radiocontrast performance, (e, f) silica coated gold nanorods theranostics agents for targeted tumor imaging and bio-distribution, (g, h) novel engineering of liposomal surface with red emissive graphene oxide flakes and their microscopic image, (i) in vivo photo transduction response under near infrared light exposure at various time points, (j) photo triggered drug delivery response of doxorubicin loaded graphene oxide-liposomal theranostics platform and (k-n) in vitro and in vivo targeted cells/tumor imaging and distribution using red emissive graphene oxide flakes coated liposomal theranostics system, (o-r) near infrared light mediated solid tumor ablation followed by chemo-photothermal therapy and compared with standalone chemotherapy using folic acid attached red emissive graphene oxide flakes coated liposomal theranostics system and (s,t) representation of a novel engineering of gold nanorods supported liposomal theranostics system, schematic and microscopic image. Reproduced with permission from ACS Appl. Bio Mater. 2019, 2, 8, 3312, Bioconjugate Chem. 2018, 29, 12, 4012 and Bioconjugate Chem. 2018, 29, 5, 1510 published by American Chemical Society.

So far, various nanoparticles have been proposed and tested for bio-imaging and therapeutic applications of cancer, but individually. Integrating these two components within a single system brings theranostics possibility. Such designs can be achieved via surface coating methodologies where imaging agents are in the core of therapeutics shell and the other way around. For example, mesoporous silica (MS), graphene quantum dots/flakes, liposomes, gold nanoparticles (GNPs), etc. are being widely used particles for cancer imaging and therapeutics individually. Combining any two materials in one system augments their overall theranostics performance due to high surface area, high cargo payload, better stability, and easy surface modification. For example, encapsulation of GNPs in deep MS shell and covering liposomal surface with graphene oxide flakes/emissive dots are current thrust area in nanomedicine and cancer theranostics. In brief, alone gold nanoparticle can perform bio-imaging application but cannot carry extra amount of cargo molecules to perform theranostics response whereas parent silica can carry high cargo molecules but cannot demonstrate stimuli responsive therapeutics named as photodynamic or photothermal therapy. Hence, the combination of these two components at nanoscale followed by molecular engineering turned out in new theranostics design. It has been noticed that the precise encapsulation of gold nanoparticles (especially gold nanorods, GNRs) in a single deep MS with high surface area (more than 900 m^2^/g) and cargo capacity (more than 40 %) at the large-scale production has been noticed as a challenging task. Based on the literature survey we have noticed that this is ongoing research in countries like India, USA, China, Japan, Singapore etc. for various applications. It has been well documented that to maintain the stability and optical property of gold nanorods (GNRs) only, thin layers of mesoporous silica (MS) are deposited over gold nanoparticles 23. The designed nanoparticles are further used for multifunctional cancer theranostics (diagnostics and therapeutics) such as contrast agent for X-ray computed tomography imaging (X-ray CT) of cancer cells/tumor and NIR responsive photothermal therapy for various cancer cell lines as shown in **Figure 5**.

It is well documented that several compositions of functional inorganic systems demonstrate unique physicochemical properties, high therapeutics efficiency, and multimodal imaging ability. Biocompatibility and degradability are still remaining concerns. To overcome these limitations several soft materials such as liposome, polymeric particles, micelles, etc. have been proposed for cancer treatment on account of their biocompatibility, aqueous solubility, and high drug loading capacity 24. Alongside, various morphologies of carbon based nanohybrids such as carbon nanodots, nanotubes, graphene oxide, graphene lipid composites etc. are being explored in medical applications such as sensing, drug delivery, gene delivery, tissue engineering etc. Major drawbacks with applications of soft and carbon-based materials are their poor stability, lack of NIR emission for deep tissue imaging and photothermal ability. On the other hand, lipid self-assembled liposomal nanoparticles have gained tremendous attention, but easy degradation is always a major concern for soft liposomes due to their fragile nature. Additionally, parent liposomes are limited with drug delivery application and reinforce their theranostics property when these platforms are functionalized with imaging and therapeutics molecules. A novel design of biodegradable NIR responsive graphene oxide nano-flakes-liposome nanocomposite has been studied for multifunctional cancer theranostics in vivo models recently. The supported emissive graphene oxide flakes (GOF) enhance the stability of fragile liposomal nanoparticles. Further, the exterior surface of graphene oxide flakes is easily available for targeting molecules functionalization named as folic acid targeting ligand for folate receptors. GOF-liposomal (GOF-Lipo) composite nanoparticles (size of ~200 nm) have been designed at ambient conditions which ensures (a) uniform coating of GOF (size of ~ 20 nm with ~ 1.5 nm thickness) over liposome, (b) uniform distribution and (c) specific architectural features that enhance their synergistic multifunctional ability. In addition, the designed system shows good aqueous solubility, biocompatibility, red emissive and NIR responsive photothermal property, specific bio-distribution, localized tumor diagnosis using single dose of nanocomposite with its successful combined chemo-photothermal therapy. The engineered theranostics system has been evaluated with better biocompatibility, stability, stimuli responsive drug delivery, targeted imaging, specific targeting ability for cancer cells/tumor, NIR responsive photothermal therapy and combined chemo-photothermal therapy for breast cancer cell lines. In their drug delivery application, the drug release response from nanocomposite is evaluated at pH 7.4 and pH 4 before and after NIR exposure. At a pH of 7.4, about 3 % of drug release is noted before NIR exposure which increases to ~ 30 % after NIR exposure. This is once again attributed to the degradation of engineered GOF-Lipo nano-composite owing to generated heat on NIR exposure. At a pH 4, a drug release of ~ 8 % is seen before the NIR exposure, while more than 40 % drug release is calculated after NIR exposure. The marginally higher release at pH4 as compared to pH 7.4 can be attributed higher disintegration of GOF-lipo composite on the combined effect of NIR and acidic pH 24.

On the other hand, a bio-responsive and degradable nanohybrid that is unsaturated red emissive carbon dots (C-dots) decorated liposomal nanohybrids has been designed and tested for localized tumor imaging and light-mediated tumor growth inhibition. Along with the emissive property, these attached QDs demonstrate heat response under NIR light exposure due to unsaturated carbonic framework. However, they also produce reactive oxygen species (ROS) that cause photo triggered cancer cell death and tumor regression. Photothermal and oxidative damage on solid tumor area have been noticed which is a major limitation of such therapeutics modality. The support of QDs helps in small molecules attachment like folic acid as targeting ligands. 200 nm of surface modified nanoparticles demonstrate better aqueous dispersibility, excellent photothermal response (62 °C in 5 minutes), good biocompatibility, and targeted cellular uptake (in 4 h). Site-selective tumor reduction without affecting the surrounding healthy tissues at minimum dose has been achieved under irradiation of 808 nm light. Next, plasmonic gold nanorods also improve the stability of soft liposomal nanoparticles. The gold nanorods supported liposomes demonstrate imaging guided photothermal therapy and chemotherapy of cancer cells. The engineered dual therapeutic gold nanorod-liposomes comprises of nanorods support and doxorubicin (DOX) anticancer chemotherapeutic drug loading. These supported gold nanorods not only serve as a photothermal agent and increase the drug release in intracellular environment of cancer cells, but also provide mechanical strength to liposomes by being decorated both inside and outside of bilayer surfaces mentioned in the **Figure 5**. The designed nanohybrid has been tested for synergistic chemo-photothermal therapy for breast cancer cell lines. In brief; prior to designing the gold nanorods-liposomal theranostics nanohybrid, gold nanorods have been prepared using cetyltrimethylammonium bromide (CTAB) as a structure-directing agent followed by seed-mediated growth procedure. Finally, the surface modified gold nanorods are introduced in the film hydration and sonication process to obtain self-assembly of lipid (1:9 mixture of dipalmitoyl phosphatidylcholine (DPPC) and 1,2-distearoyl-sn- glycero-3-phosphocholine (DSPC) bilayers and surface modified nanorods that form the gold nanorods supported liposomal theranostics platform with ~170 nm size. In the photothermal transduction experiment, designed nanohybrid hyperthermia temperature (43 °C) in 5 min of NIR exposure time for 0.1 mg/mL of concentration. Further, better response in the temperature was recorded in 3 min for 0.2 mg/mL concentration and 53 °C in 5 min for 0.5 mg/mL at 1 W power.

Next, it should be noted that better colloidal stability, better blood circulation time, specific biodistribution and specific binding ability of any nanoparticles are essential parameters prior to use them in targeted cancer theranostics applications. These properties could be improved by coating the surface of nanoparticles with polyethylene glycol (PEG) known as PEGylation which reduces or hamper the adsorption of blood proteins over the surface of circulated nanoparticles in the blood stream. PEGylation makes nanoparticles stealth which is a widely explored approach in the area of nanomedicine. In 1977, PEGylation for drug delivery applications has been reported for the first time and in 1990 FDA approved the first PEGylated protein product for immunodeficiency disease. Overall, about 8 PEGylated protein therapeutics have been approved by FDA for biomedical applications. In the case of nanoparticle therapeutics, Doxil® is the first FDA approved PEGylated liposomal nanoparticle in 1995 which is loaded with anticancer drug doxorubicin. Doxil® demonstrates better bioavailability (90-folds) compared to free drug with a drug half-life of 72 h and circulation half-life of 36 h in one week of post-injection. Interestingly, PEGylation protects the surface of nanoparticles from opsonization and phagocytosis with better dispersion after systemic administration. It should be noted that PEGylation improves systemic blood circulation time and reduces the immunogenicity of the injected nanoparticles. Conjugated PEG chains and density improve the hydrophilic nature of nanoparticles that make hydrated cloud over nanoparticles core with a large omitted volume that strongly prevents the adsorption of blood components and proteins on the nanoparticle's surface. However, covering, density and molecular weight of PEGylated molecules alter the physicochemical and biological properties of nanoparticles.

## Nanovalved theranostics systems

**Figure 6:**
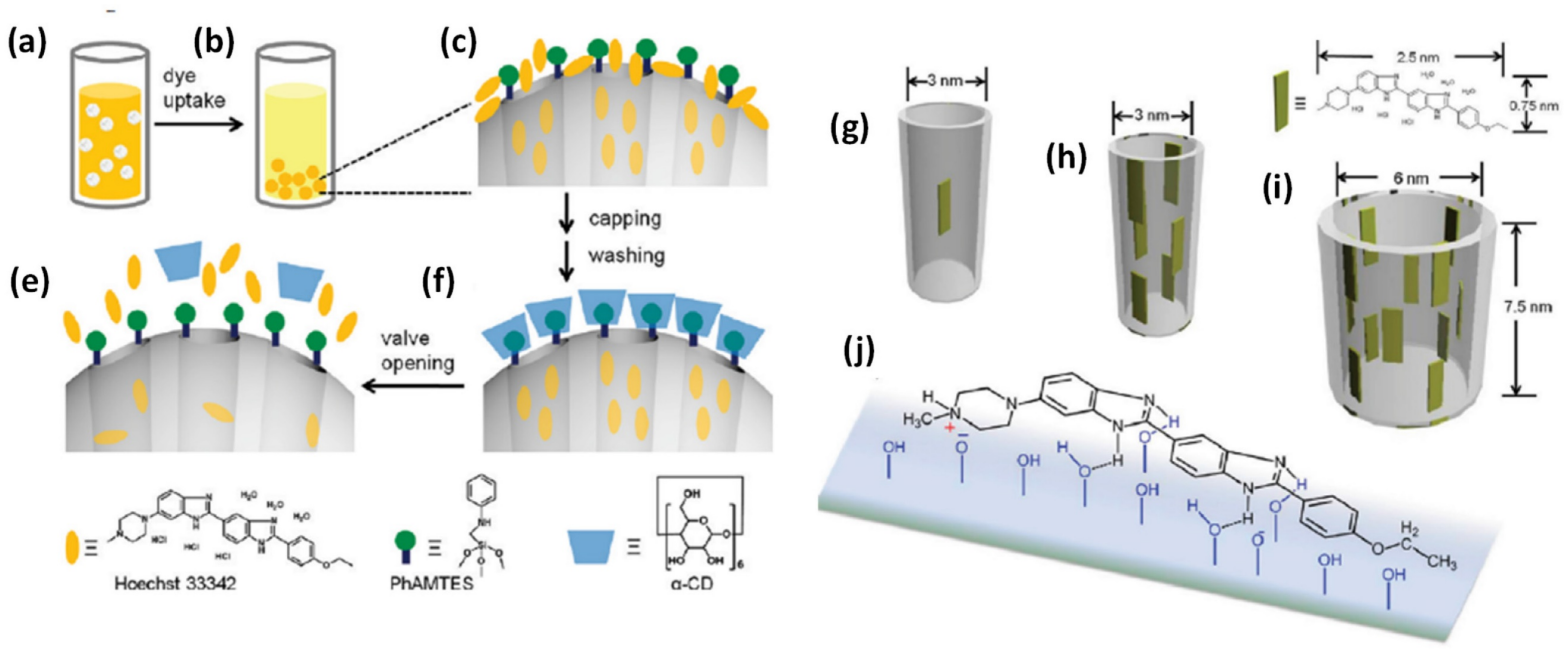
Schematic illustration of nanovalve-gated multifunctional mesoporous silica theranostics systems for loading and release of Hoechst 33342 molecules. (a,b) loading of Hoechst 33342, (c) zoomed particle's surface before capping and washing, (d) loaded Hoechst 33342 molecules in pores after capping and washing, (e) release of Hoechst 33342 molecules from the pores, (g-i) loading of dye molecules in different pore sizes of the nanoparticles and (h) chemistry of Hoechst 33342 molecule adsorbed on the silica surface. Reproduced with permission from J. Phys. Chem. C 2011, 115, 19496 published by American Chemical Society.

Premature leakage of loaded cargo molecules (imaging and therapeutic agents) from the designed nanotheranostics platforms is always a major issue. To prevent such premature release prior to reaching the target site, nanovalves/or nanogates have been decorated on the exterior surface of the theranostics where pores are opened. On the other hand, the nano-gated concept in theranostics systems has been evolved in the area of nanomedicine, where stimuli responsive controlled drug release can be triggered for improved and safe cancer therapies through zero or negligible premature release 75. Nano-gates seal the pore opening of nanohybrid/theranostics to prevent the premature release of drug 75,80. Surface modifications with various functionalities is used for controlled the drug release ass depicted in **Figure 6** 75,80,81. Pore are gated using bulky groups like supramolecules (nanovalve of cyclodextrin, aromatic amine, polymers, proteins, support of lipid layers) or nanoparticles, such as, carbon quantum dots, gold nanoparticles, iron oxide nanoparticles, etc 75,81. These sealed gates either degrade or get detached on exposure of specific stimuli or to the cancer mimicked environment 81. To date, various structures based nanohybrids such as light responsive plasmonic nanoparticles, superparamagnetic nanoparticles with responsiveness to magnetic fields, polymeric and liposomal based nanohybrids which are high-frequency ultrasound responsive, etc., have been thoroughly studied for remotely triggered drug release 75,81.

Another recent development in theranostics viz., a nanovalve gated mesoporous silica (MS) with stimuli responsive controlled drug delivery has required great attention. The exterior surface of MS is functionalized with other pore covering agents such as metal nanoparticles, graphene quantum dots, carbon nanodots etc. for preventing the premature drug leakage and for various stimuli responsive controlled drug delivery 75,81. In that context, Sir F. Stoddart and J. Zink have conceptualized stimuli responsive supramolecular gatekeepers on mesoporous silica nanoparticles that respond to various stimuli such as light, temperature, pH and enzyme 81,82 among which individual stimuli, pH and redox-responsive release mechanisms have been recognized as effective ones 81.

## Stimuli responsive mesoporous silica

Various MS gated nanohybrids have been exploited for pH-responsive controlled drug delivery in cancer mimicked environment (pH ~ 2-4) 82,83. MS as a cargo carrier is reported by Vallet-Regi et al. in 2001 for the first time 84. Stimuli responsive release of guest molecules from MS is reported by Mal et al. in 2003 85. The MS nanoparticles are prepared by sol-gel process and N-(3-triethoxysilylpropyl-2-aminoethyl)-ethylenediamine is grafted on its exterior surface to obtain amine functionalized mesoporous nanohybrid 75. In detail, anchored amine groups are deprotonated at high pH values and the delivery system goes to an “open gate” state 75,82. However, at low pH conditions, amine groups repel to each other due to coulombic repulsion effect and cover the pore openings brining the delivery system to a “close gate” state 86.

In 2004, Sir F. Stoddart, J. Zink and coworkers have designed nano-gated mesoporous silica utilizing the recognition between surface-tethered naphthalene-containing secondary dialkylammonium ions and supramolecular gates (dibenzo[24]crown-8 (DB24C8) 86. The loaded drug molecules from the pores of MS are released at various pH levels and the rate of cargo release is determined by the effect of electrostatic interactions and steric hindrance of the added salts. Similar concept is described by Kim and coworkers in 2007, where the pores are blocked by grafting a biocompatible version of pH-responsive polyethylene amine (PEI)/ cyclodextin (CD) polypseudorotaxanes 87. In 2008, Sir Stoddart and Zink's groups have reported the first cucurbituril based supramolecular nanovalves on MS nanoparticles (bisammonium stalks and cucurbit 6 uril (CB6) rings) 88. The successful operation of these nanovalves on mesoporous nanohybrids is observed under pH variation. The nanovalves operation depends on the ion-dipole interaction between CB6 and the bisammonium stalks as a function of pH values of an external environment. When pH values are neutral and acidic, CB6 rings close the bisammonium stalks tightly and results in the pore sealing of nanohybrid. However, the deprotonation of bisammonium stalks is noticed in basic environment (pH ~ 9-10). Hence, the pH-responsive supramolecular nanovalves play a significant role in cancer therapy due to the variation in pH values in cancer and normal cells in living systems.

**Figure 7:**
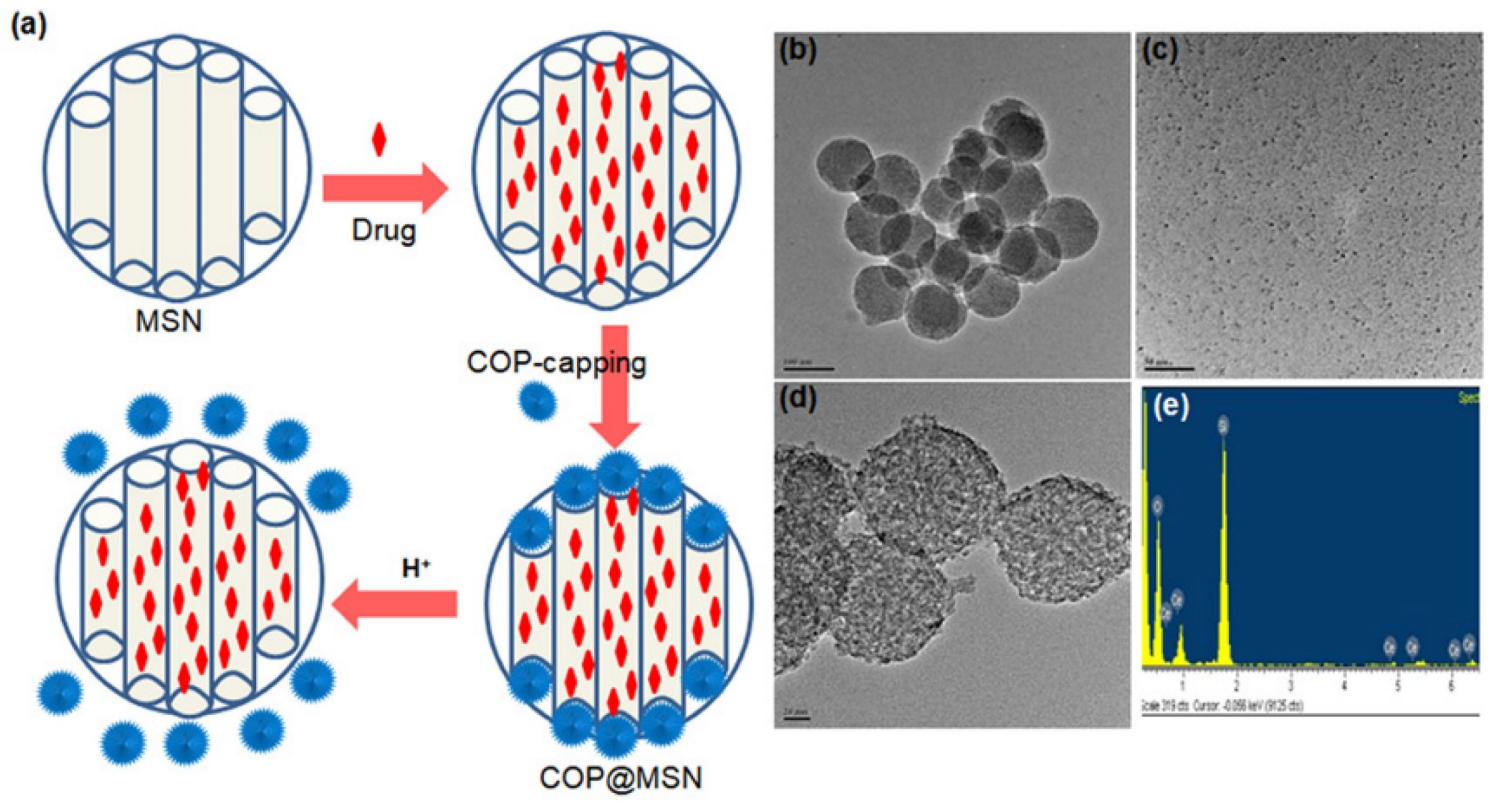
(a) Schematic representation of nanoceria capped drug loaded mesoporous silica for pH-triggered release with microscopic and elemental understanding. Reproduced with permission from ACS Appl. Mater. Interfaces 2019, 11, 1, 288 published by American Chemical Society.

Zink and coworkers have contributed significantly to studies on the pH-responsive cargo uptake and release in cancer mimicked environment using cyclodextin (CD) threaded phenylaminomethyl stalk based nanovalves as reported in 2011 89. It is shown that, the CDs encircle the stalks on the nanohybrid surface through hydrophobic interactions. On protonation in the stalks, CD gates/caps detach from the stalks and result in the pore opening in acidic condition (pH ~ 3-5). Similarly, other types of pore covering agents such as poly (methacrylic acid) (PMAA), lipid bilayer, metal complexes etc. are incorporated on the exterior surface of MS nanohybrids 86,90. However, their poor aqueous solubility is a major concern for nanomedicine with the exception of few molecules such as lipid bilayer, CB (6) etc 90. Recently (in 2018), cerium oxide nanoparticles gated mesoporous silica hybrids has been studied for pH trigger control drug release and intracellular drug delivery of anticancer drug doxorubicin 90. Pore opening of carboxyl-functionalized mesoporous silica particles are gated with aminated cerium oxide nanoparticles (COP) where these pores are opened in acidic conditions to release the loaded drug as shown in **Figure 7**.

**Figure 8:**
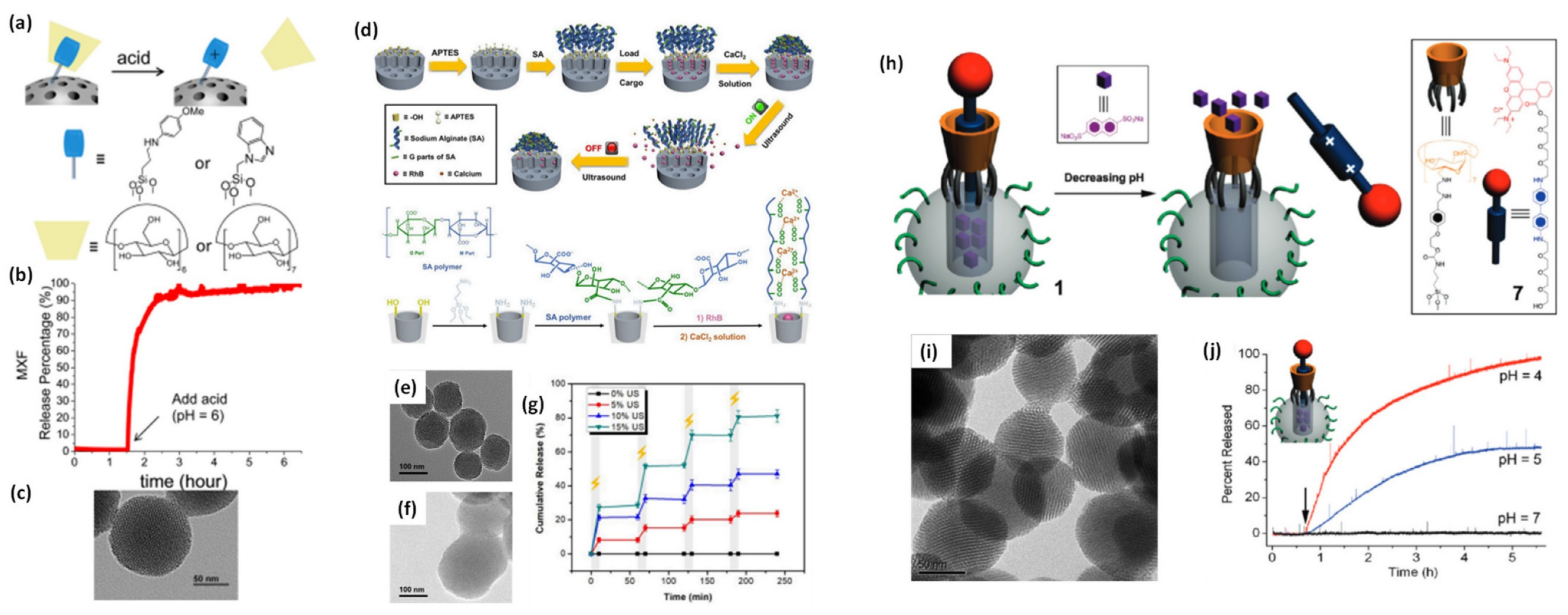
Schematic illustration of stimuli responsive nanovalve-gates on mesoporous silica nanoparticles. (a-c) cyclodextrin gated mesoporous silica surface and their cargo release pattern reproduced with permission from ACS Nano 2015, 9, 11, 10778 published by American Chemical Society, (d-g) cross-linked network on mesoporous silica nanoparticles and molecular design of sodium alginate (SA) along with release kinetics and microscopic images, reproduced from Front. Chem. 7:59. doi: 10.3389/fchem.2019.00059 an open-access article under the terms of the Creative Commons Attribution License (CC BY), (h-j) nanopiston mechanism of phosphonate covered mesoporous silica through acid-cleavable acetal bond, reproduced with permission from J. Am. Chem. Soc. 2010, 132, 37, 13016 published by American Chemical Society.

Alternatively, more soluble, biocompatible and stable systems such as gold nanoparticles, graphene quantum dots/carbon quantum dots (GQDs/CQDs) are being recently explored as gate keepers (2013, 2014 and 2016) 75,91. In a first such report in 2013, Qu and coworkers have attempted to cap the exterior surface of MS by CQDs and significant premature release (about 15 %) of anticancer drug, doxorubicin (DOX) is observed 92. The issue of premature release of cargo molecules from MS has been later addressed to a larger extent by Fu and coworkers in 2014 using graphene quantum dots (GQDs) as caps 93. No other literature is available on the design and testing of GQDs/CQDs as capping agents. Thus, there appears to be a huge scope for further improvements in pore sealing, prevent premature drug release and aqueous solubility of MS based systems capped by the above agents. These limited reports further suggest that the inconsistency between pore diameter of MS and size of CQDs/GQDs results in loosely sealed pores attributing to the premature drug release. A part of the present thesis attempts to discuss a successful design of a MS based bio responsive multifunctional theranostics system with tightly sealed pores by fluorescent CQDs/GQDs 91. The concept of fluorescent nano-gate on mesoporous silica and its controlled drug delivery response in cancer mimicked environment is reported (2016) as a consequence that solves the solubility problem and sophisticated stimuli responsive mechanism of macromolecular nanovalve for drug delivery 91. Mechanized mesoporous silica nanoparticle with supramolecular nanovalves have been widely studied for controlled cargo/drug release response as shown in **Figure 8.** This is believed to provide a new direction for stimuli responsive nanomedicine for cancer theranostics.

Next, many diseases are related to redox homeostasis in the human body and cancer being the deadliest one 88. Importantly, redox homeostasis is also responsible for atherosclerosis, heart diseases and chronic diseases of major internal organs. Thus, it is significantly important to design a redox-responsive cancer theranostics system for the intracellular transport of drugs. Further, the continuous progress and successful therapeutic outcomes in redox responsive nanohybrids are reported. In 2003, Lin and coworkers have designed cadmium sulfide (CdS) decorated MS nanoparticles as a first redox-responsive drug delivery system 94. In detail, the pores of drug loaded MS are covalently capped by mercaptoacetic acid functionalized CdS nanocrystals through 2-(propyldisulfanyl) ethylamine linker. Prevention of premature drug release from this system for a long time has been observed under normal physiological conditions. However, the disulfide linkages between the MS surface and CdS nanoparticles could be cleaved in the presence of various disulfide-reducing agents, such as glutathione (GSH, 2-20 mM), dithiothreitol (DTT) and mercaptoethanol (ME) 82,91,94. Along the same lines, Lin and coworkers have also designed superparamagnetic iron oxide nanoparticles (Fe_3_O_4_) gated MS nanohybrid as a stimuli-responsive controlled drug delivery system 95. The disulfide linkages between the MS surface and Fe_3_O_4_ nanoparticles could be cleaved with above mentioned disulfide-reducing agents to implement successful redox-responsive controlled drug release. The above mentioned site-specific magnetic nanohybrids can also be attractive for the development of targeted and controlled drug delivery in intracellular environment opening a new direction for site-selective controlled drug delivery devices and nano biosensors.

**Figure 9:**
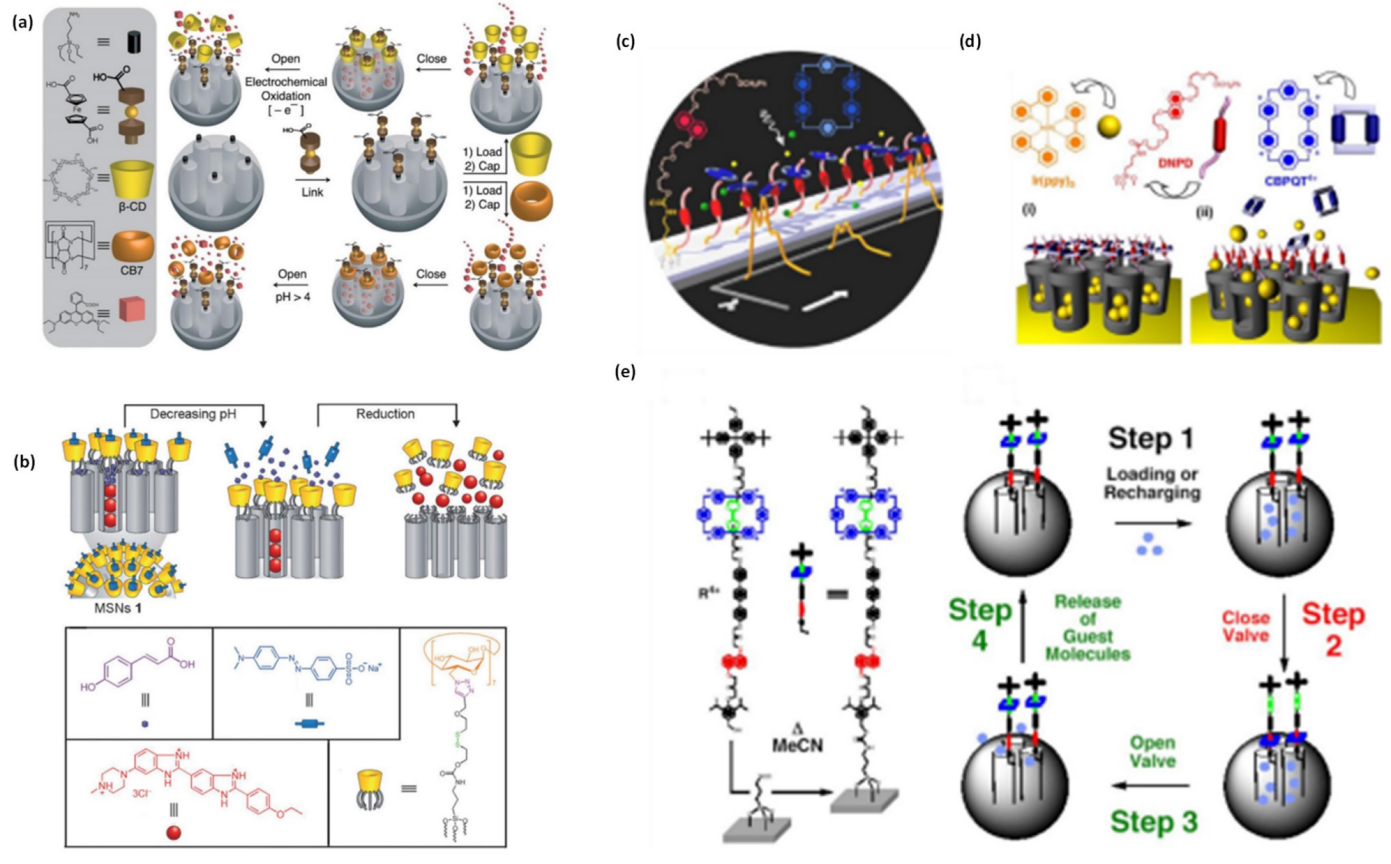
(a-e) Schematic illustration of operation of nanovalves gating the pore openings on silica particles in various stimuli conditions reproduced with permission from Theranostics 2019, 9, 3341, doi: 10.7150/thno.34576 open access article under the terms of the Creative Commons Attribution (CC BY-NC) license published by IVYSPRING.

Zink's group developed macromolecule based redox responsive gatekeepers in 2004 86. These gates are related to tethered 1, 5-dioxynaphthalene (DNP)-containing derivative (DNPD) that is decorated on the MS surface and cyclobis-(paraquat-p-phenylene) (CBPQT4^+^) which identify the DNP units through non-covalent interactions. The specific signal for pore opening is from an external reducing reagent (NaCNBH3) which operates the nanovalve and permits the control release of cargo molecules. Subsequently, the pseudorotaxane based gatekeepers are developed by same group through a fabrication of a bistable, reversible redox-responsive nano-switch on MS exterior surface 96. The nano-switch is controlled with mild redox reagents and relied on the sliding of gated rings on the bistable thread. A polymeric gated MS based redox system is reported by Feng et al. in 2008 for the first time 97. The cross-linked polymeric network on MS surface is worked as a redox responsive nano-switch where poly (N-acryloxysuccinimide) is attached on the exterior of MS pore through cystamine linking a disulfide-based bifunctional primary amine that could respond in GSH, DTT, ME, environment as explained in **Figure 9**. In 2009, Wang et al. has reported aptamer functionalized polyelectrolyte multilayers (PEM)-coated MS nanohybrids as a targeted controlled drug delivery system 98. These layers cover the MS pore opening and deliver the drug in cytosolic space. Cyclodextrins are also preferred to modify the exterior surface of MS with the various disulfide linkers that could prevent the premature release of the loaded drugs but leading to controlled drug delivery performance in the cytosolic space 98. In 2012, Li et al. introduced GSH responsive disulfide bond containing PEG modified MS nanohybrid as controlled drug release system 99. In summary, the redox-responsive gates on the surface of MS based nanohybrids are widely explored in controlled drug delivery applications.

On the other hand, the above two discussed stimuli responsive systems are basically designed for controlled drug delivery in intracellular environment. A nano-gate operation on MS surface could also be controlled through external stimuli viz., light exposure and these systems are in high demand as controlled drug delivery devices. Light irradiation can control the distribution and release of drug molecules from cargo carriers in cancer mimicked environment. The generated heat by photothermal agent during light exposure operates the nano-gates on MS surface. Tanaka and coworkers reported the light responsive coumarin modified MS nanohybrid for the first time 100. Designed system reveals the controlled release of drug via light responsive reversible intermolecular dimerization of coumarin derivatives on MS surface. Photosensitive coumarin blocks the MS pores through photodimerization of cyclobutane dimers under 310 nm light exposure. The cleavage of cyclobutane rings is observed during 250 nm UV light exposure that led to the pore opening of MS nanohybrid. Interestingly, various stalk and ring components (pseudorotaxanes approach) are also applied to understand the control drug delivery with light. In 2009, for the first time, Sir Stoddart and coworkers fabricated a controlled drug delivery vehicle that is based on photo responsive pseudo rotaxane of azobenzene (AB) derivative and *β*-cyclodextrin (*β*-CD) 101. The exterior surface of MS is grafted with pseudo rotaxane stalks viz., 4-(3-triethoxylsilyl propylureido) azobenzene (TSUA) groups or more water-soluble (E)-4-((4-(benzylcarbamoyl) phenyl)diazenyl) benzoic acid groups. Under the irradiation of 351 nm light, both azobenzene derivatives isomerize from the more stable *trans* form to a less stable *cis* form and then *β*-CD or fluorescently labeled pyrene-*β*-cyclodextrin (Py-*β*-CD) are introduced. The strong binding ability between *trans*-AB and *β*-CD locks the *β*-CD rings of MS surface. On the other hand, owing to the weak binding affinity between *cis*-AB and *β*-CD, the isomerization of *trans*- to *cis*-AB stalks leads to the dissociation of pseudorotaxanes and thus allowing the release of cargo molecules from carrier system. These CD based gates are applied by several groups after the demonstration by Zink and Sir Stoddart 88,90,96.

Photosensitive cleavable thymine-based nano-gates are conceptualized in 2012 where 365 nm UV light irradiation results in the formation of cyclobutane dimer and then block the pores whereas on a 240 nm light irradiation, the pores are opened due to photocleavage of cyclobutane dimer and entrapped drug is released from the carrier system 102. Similarly, continuous progress has been reported on light mediated controlled drug delivery by various nanohybrids such as, up-conversion nanohybrid, silica coated gold nanorods, NIR dye loaded lipid, silica, polymeric nanoparticles, etc. The NIR responsive metal nanoparticles (800-900 nm wavelength) encapsulated MS nanohybrids for near-infrared (NIR) light mediated controlled drug delivery is a recent development in nanomedicine and these studies are reported from 2010 onwards 16-18,103. A noteworthy report in this context demonstrate an azobenzene gated mechanism by using MS coated NaYF4:Tm, YbNaYF4 nanohybrids under 980 nm light exposure 104. The released drug amount could be controlled through varying the intensity or exposure duration of NIR light 103.

Several other NIR active systems with internal photothermal heating by plasmonic nanoparticles, photodimerizations (*cis-trans* conversion) have been reported for controlled release of drugs or dyes 106. Focus has been on designing nano-gated light mediated systems for site specific localized treatment avoiding the irradiation of surrounding healthy tissues 75,82. In 2014, MS coated gold nanorods gated with sulfonatocalix[4]arene (SC[4]A)/quaternary ammonium supramolecular switches have been designed as a novel cancer theranostic platform 103.

Through a supramolecular host-guest interaction these nanovalves close the pore opening of the nanohybrid. The internal heating generated by gold nanorods under NIR light exposure decreases the host-guest binding affinity so that SC[4]A rings dissociate from the stalks on the exterior surfaces thereby allowing the pore opening for release of trapped cargo molecules.

Among the light responsive techniques, UV light responsive method shows various side effects on healthy tissues due to its poor penetration ability 107. NIR light responsive controlled drug delivery systems have several advantages over the above as well as the other stimuli responsive approaches. Controlled switching gated operation for controlled delivery, low side effects, specific distribution of drug molecules in site location, etc are few of the advantages 34,64. Additionally, these find significant importance in nanomedicine due to its ability to deeply penetrate without tissue damage and its good photothermal response for controlled delivery of drugs through open and close gated mechanism 105,108.

In a living body the presence of tumors, inflammation and any type of infection can cause an increase in the temperature of the whole body by up to 4-5 °C. Hence, it is advantageous to design a temperature responsive drug delivery system that only releases drugs at temperatures above 37 °C 109. The modification of MS surface with temperature responsive nano-switch makes it to be a stimuli responsive controlled drug/dye delivery system. So far, poly-N-isopropylacrylamide (PNIPAM) and its derivatives are widely used temperature sensitive gatekeepers on MS surface 109. More recently, Yang's group has designed the thermo responsive core-shell magnetic MS nanohybrid where Fe_3_O_4_ nanoparticles are in cores and the exterior MS surface is functionalized with cross-linked thermosensitive (PNIPAM, PNIPAM-co-NHMA) copolymer 110. A controlled drug release is noticed below the lower critical solution temperature (LCST) of these polymers where they exhibit a hydrophilic extended state. PNIPAM is functionalized on the internal surface of the MS through atom transfer radical polymerization (ATRP) as reported by Lopez and coworkers, who claimed that the PNIPAM-functionalized MS can release the drug molecules at a high temperature (50 °C) and hinder the drug release at low temperature (25 °C) 109. A thiol-functionalized polymeric gated MS surface with pyridyl disulfide-terminated poly (N-isopropylacrylamide) (PNIPAM-S-S-Py) is examined by Oupický et al 111. The polymer gate is in the random coil formation at room temperature (below the LCST of the polymer) which results in the release of loaded cargo molecules at the same temperature.

Interestingly, Wang, Cui and coworkers have reported dual stimuli responsive (pH and thermal) copolymer-lipid bilayer-coated MS drug delivery system in 2013 112. The designed system contains natural phospholipids (soyphosphatidylcholine, SPC) and poly(N-isopropylacrylamide-methacrylic acid-octadecyl acrylate) copolymer (p(NIPAM-MAAODA)) with a phase transition temperature of 42 °C and maximum DOX release (54 %) is observed at 42 °C that is five times larger with compared release at 25 °C temperature. Similarly, many more temperature responsive nano-gated systems have been designed and their successful controlled drug release performances are reported by several research groups 113.

## State of art in nanotheranostics area

Various nano sized formulations for biomedical applications are known. Several multimodal imaging agents and therapeutic probes are well studied for molecular imaging and therapies. These imaging and therapeutic systems for medical applications are well explored individually. Among them, a few have reached clinical trials, but are struggling with FDA approval. Stimulatingly, selective surface engineering can influence the specific design of theranostics systems which make them suitable for targeted diagnosis and therapeutics in pre-clinical and clinical models. On the other hand, reliability and reproducibility are major issues which can be resolved by using numerous synthesis methods such as top-down, sol-gel, solvothermal, hydrothermal, surfactants assisted, seed mediated growth, post-graft, etc. In fact, it is the choice of synthesis method which can produce a multifunctional theranostics system with controlled sizes and shapes. The role of various characteristics such as, size, shape, surface chemistry, framework, composition, physicochemical property, etc. of these systems on the theranostics performance have been understood by using modern techniques.

Although the chemistry and biomedical applications of various nano sized systems are known since past few decades, there are several major concerns that need to be answered/addressed such as:

1. Knowledge on how to control the size and shape of nano sized imaging and therapeutic agents is not fully understood. Their surface chemistry and boundary between inorganic core and stabilizing agents/capping agents at nanoscopic level are poorly understood so far. For example, the organic soft moieties on metal surface and their architecture are not visualizable through microscopic techniques (TEM, SEM, AFM, STEM etc.).

2. The formation of specific size distributed nanoparticles such as gold nanoparticles (nanorods), mesoporous silica, graphene oxide flakes on liposomal surface, quantum dots on liposomal surface, gold nanorods on liposomal surface, etc. their mechanism and surface chemistry are less understood.

3. Similarly, the various modes and details of the interaction between two (metallic and nonmetallic) components are not known fully.

4. The nanoparticle's cross talk with cancer cells is less understood.

5. Rapid and scalable production of imaging and therapeutic systems with high product yield is highly challenging.

6. Understanding the theranostics performance (specific site location, high cargo capacity, photothermal conversion efficiency, etc.) of multifunctional systems is inadequate but important.

The present article covers the review of progress and recent developments in nanotheranostics for targeted diagnosis and treatment of cancer. So far various organic and inorganic hybrid nanomaterials have been tried for multifunctional cancer theranostics applications. However, biodegradation, biocompatibility, aqueous solubility, photostability, low cargo capacity, poor theranostics performance are major concerns in nanomedicine. This review represents a step towards the choice of advanced multifunctional theranostics systems for cancer diagnosis and therapeutics. Various parameters related to nanotheranostics such as rapid and scalable design of nanohybrids, biodegradation ability, enhanced biocompatibility, high surface area, high cargo capacity, enhanced theranostics performance, good water solubility, specific targeting ability as well as bio-distribution have been addressed. This includes the attempt for a simple design of multifunctional nanohybrids by integrating multimodal diagnostics agents (X-ray CT and fluorescence), chemotherapeutic anti-cancer drug) and photothermal agents in a single system. In addition, the issues of surface modification, their role on enhanced theranostics performance and combined chemo-photothermal therapeutic efficiency of various designed systems are addressed.

For example, plasmonic gold nanorods are prepared by using CTAB surfactant as a structure directing agent, but its shows high toxicity. Hence, the direct use of gold nanorods are prohibited in cancer theranostics applications. Therefore, silica coating has been proposed to overcome this limitation, but the effective distribution of nanorods in silica is a major challenge. On the other hand, scalable and rapid process with highest surface area and cargo capacity and preventing premature release of loaded cargo are another demanding parameters for gold-silica based nanotheranostics. The prevention of premature release of loaded cargo and their stimuli responsive controlled release have been achieved nano-gates which are decorated on the exterior surface of mesoporous silica particles. Now, major concern is biodegradation, therefore, surface engineered liposomal systems have been proposed as biodegradable theranostics. There is ample scope of further research and development in fabrication of nanotheranostics systems having complete biodegradability in a given time frame and having all the above desired properties. More importantly these desirable multifunctionalities have been discussed in this article.

## Nanoparticles for solid tumors “entry and exit”

After successful design of nanoparticles or nanotheranostics, their interaction with cancer cells and solid tumors have become essential for pre-clinical and clinical trials 114. Over the years, nanoparticles and solid tumor theranostics have opened various questions regarding the interaction, biodistributions, site specific binding, easy penetration and strong retention, tumor entry and exit, and biodegradation 115,116. Enhanced permeability and retention (EPR) effect is the central dogma for nanoparticles accumulation in tumor environment. The concept of nanoparticles entry or accumulation followed by passive way in tumor microenvironment was established in 1986 by Jain and Maeda's groups. Diffusion is a widely accepted mechanism for nanoparticle's entry and distribution in tumors. 0.7% of the injected dose of nanoparticles has been reported in the literature in case of their entry in solid tumors 117. Moreover, nanoparticle's ability for passing through blood vessels into the tumor microenvironment indicates towards their successful entry into solid tumors. Surface engineering, size, shape, charge, stimuli interventions, saturating macrophage and Kupffer cells can improve their entry or accumulation but not significant 118. So far, researchers have designed nanoparticles for passive entry into solid tumor blood vessels through open gaps (up to 2000 nm) between endothelial cells which can be achieved by altering the nanoparticle size and shape 119. However, nanoparticle's transportation into the tumor blood vessels through the endothelial cells has also been reported recently 119. It should be noted that there are 26 gaps among 313 blood vessels throughout the solid tumor environment. Moreover, interfacial understanding between nanoparticles and the tumor endothelium can give us clarity on the entry mechanism which can also answer for whether all endothelial cells involved in nanoparticle's transportation. Recently, warren and Weissleder's group explained that tumor-associated macrophages uptake nanoparticles upon passing through the tumor blood vessels and distribute them in the tumor microenvironment 120. They have noticed that tumor-associated macrophages aggressively migrate toward nanoparticles belched from the leaky blood vessels and engulfed them for further redistribution in heterogeneous tumor microenvironment. Remarkably, it has been investigated that these macrophages can carry nanoparticles 2-5 times deeper in the tumor than passive diffusion. But, the question is how many nanoparticles will they transport? Further, these macrophages respond to nanoparticles according to their size 120. On the other hand, lymphatic vessels within or surrounding the tumor help nanoparticles exit from the tumor environment which depends on the nanoparticle size 121. Recently, it has been reported that exit nanoparticles from the tumor again returned to the blood system which allow them to recirculate and interact with the solid tumor for the second pass.

## Conclusion and future perspective

In summary, various examples of surface engineered nanoparticles (metallic and nonmetallic) which received tremendous attention for targeted imaging, multimode therapeutics and drug delivery have been discussed here. Further, the clinical relevance of nanoparticles-based cancer theranostics systems with current demand have been highlighted in detail. However, choosing metallic or nonmetallic components for engineering nanotheranostics agents is an always concern. Herein, we have reviewed various design of theranostics nanoparticles such as gold nanoparticles (rod shaped), silica particles, liposomal hybrids, graphene oxide flakes, quantum dots etc., followed by surface coating and nanovalved approaches. For example, graphene oxide flakes-liposomal theranostics has been designed through surface coating method and tested for imaging and anticancer activity in in vivo tumor models. On the other hand, quantum dots/or nanoparticles decorated mesoporous silica theranostics have been designed through nanovalved method and tested for targeted imaging and therapeutics of cancer. Engineered systems from both approaches demonstrate significant theranostics response in pre-clinical models. However, silica based theranostics system suffer with slow biodegradation and metabolism whereas graphene oxide flakes-liposomal theranostics face major issues of complicated engineering, uncontrolled drug release and rapid degradation. Hence, it is a major concern on the choice of theranostics system for pre-clinical validation. Hence, the choice of nanoparticles for theranostics engineering and associated challenges have been highlighted in the present article. In addition, the key requirements to design multifunctional theranostics and its limitations have also been highlighted here. Further, the various designs of stimuli (viz., redox, light, pH, thermal, etc.) responsive nano-gated systems with various supramolecular and molecular switches are reviewed in detail. Whole a significantly remarkable progress and contribution towards biocompatible design of multifunctional nanohybrids for cancer theranostics is visible, several issues on their successful parameters along with minimal cytotoxicity are yet to be understood and resolved. Overall, we have mentioned detailed information on understanding the design of multifunctional nanohybrids for cancer diagnosis and therapeutic applications. So far, iodinated contrast agents are commonly used imaging probe for diagnosis, but these contrasts show several allergic issues, nephrotoxicity, rapid clearance (less than 10 min.), nonspecific bio-distribution, lack of surface functionalization etc. However, these contrast agents require permanent replacement with some good biocompatible contrast agents. To overcome this issue various shapes of gold nanoparticles are heavily used as contrast agent for diagnosis and their good biocompatibility is recognized recently. Due to specific optical property, good contrast ability (brightness and radio density) these gold nanoparticles can be applicable for human trials in the near future.

On other side, mesoporous silica (MS) has been considered as a safe material for drug delivery due to its high surface area, high cargo capacity, easy to surface functionalization, good biocompatibility etc. The MS based carrier system can deliver the diagnostics and therapeutics agents at the targeted site which can solve the problem for specific distribution of several drug molecules and specific tumor diagnosis. Due to good biocompatibility of silica, few silica based nano sized hybrids are in clinical applications. Thus, the chances of MS can be approved for human trials because of its high cargo capacity, biocompatibility, and Sir F. Stoddart's (Nobel Prize in Chemistry, 2016) and J. Zink's nano-gated concept for stimuli responsive controlled drug delivery at targeted tumor sits. In addition, several formulations of soft materials are in clinical trials (liposomal nanohybrids with drug encapsulations) but these systems are far from their multifunctional performance. Further, near-infra red (NIR) light responsive photodynamic therapy is known for clinical applications. However, biodegradation, red emissive nature, NIR responsive, multifunctional ability of nano sized hybrids materials can be considered for new demand for clinical practice. Moreover, here a new design of red emissive and NIR responsive multifunctional nano sized system for cancer theranostics applications has been discussed. The designed system is further biodegradable in nature under therapeutics command. Thus, the designed nanocomposite can be considered as clinical relevant material for further Food and Drug Administration (FDA) approval and its clinical applications. Finally, the impact of nanotechnology on cancer diagnosis and therapies has been covered in this article. The designed each nanohybrid/composite system has advanced concept for new developments in nanomedicine and shows their capability for cancer nanomedicine and can solve the various issues of oncology.

## Figures and Tables

**Table 1:** Nanoparticle formulations and their clinical status 9,11

Nanoparticle formulation	Applications	Status
Technetium-99m rhenium sulfide colloid	Bio-imaging	Approved in European countries
Iron oxide nanoparticles	Bio-imaging	Approved by FDA 1996
Hafnium oxide nanoparticles	Therapy	Clinical trials
^64^Cu labelled liposome loaded with doxorubicin	Cancer Theranostics	Phase I (2017)
^64^Cu labelled polyglucose nanoparticles	Bio-imaging	Phase I (2021)
^124^I or ^89^Zr labelled silica nanoparticles	Bio-imaging	Phase I (2022); NCT01266096, Active (2021)
NIR fluorophore tagged RGD-silica nanoparticles	Bio-imaging	NCT02106598, Phase I/II, Recruiting (2022)
Silica-gold core-shell	Therapy	NCT00848042, Completed (2017) NCT02680535, Completed (2021)
PEGylated gold nanoparticles	Targeted therapy	Phase I
Nucleic acids conjugated gold nanoparticles	Therapy	NCT03020017, Phase I, Completed (2020)
Aminosilane-SPIO nanoparticles	Therapy	Phase I
CdS/ZnS quantum dots	Bio-imaging and Therapy	Phase I
^89^Zr labelled polymeric nanoparticles loaded with docetaxel	Bio-imaging and Therapy	NCT03742713, Phase II, Completed (2020)
Fluorophore tagged organic micelle	Bio-imaging and Theranostics	NCT03735680, Phase II, Completed (2022) NCT05048082, Phase II, Recruiting (2022)
Albumin colloid	Bio-imaging	Clinically approved
Lipo-Dox (Taiwan Liposome)	Therapy	Clinically approved
Liposomal alendronate	Therapy	Under clinical trial
Doxorubicin liposomes Doxil (Janssen products)	Therapy	Phase II
Gd-polysiloxane nanoformulations	Bio-imaging and Theranostics	Under clinical trial
